# Vertical 3D Nanostructures Boost Efficient Hydrogen Production Coupled with Glycerol Oxidation Under Alkaline Conditions

**DOI:** 10.1007/s40820-023-01150-1

**Published:** 2023-07-29

**Authors:** Shanlin Li, Danmin Liu, Guowei Wang, Peijie Ma, Xunlu Wang, Jiacheng Wang, Ruguang Ma

**Affiliations:** 1 Key Laboratory of Advanced Functional Materials, Ministry of Education, Faculty of Materials and Manufacturing, Beijing University of Technology, Beijing, 100124 People’s Republic of China; 2 The State Key Laboratory of High Performance Ceramics and Superfine Microstructure, Shanghai Institute of Ceramics, Chinese Academy of Sciences, Shanghai, 200050 People’s Republic of China; 3 School of Materials Science and Engineering, Suzhou University of Science and Technology, 99 Xuefu Road, Suzhou, 215011 People’s Republic of China; 4 School of Materials Science and Engineering, Taizhou University, Taizhou, 318000 People’s Republic of China; 5 Hebei Provincial Key Laboratory of Inorganic Nonmetallic Materials, College of Materials Science and Engineering, North China University of Science and Technology, Tanshang, 063210 People’s Republic of China

**Keywords:** Hydrogen evolution reaction, Glycerol oxidation reaction, Oxygen evolution reaction, Flow cell, Nanostructure

## Abstract

Hydrogen production from electrolytic water is an important sustainable technology to realize renewable energy conversion and carbon neutrality. However, it is limited by the high overpotential of oxygen evolution reaction (OER) at the anode. To reduce the operating voltage of electrolyzer, herein thermodynamically favorable glycerol oxidation reaction (GOR) is proposed to replace the OER. Moreover, vertical NiO flakes and NiMoNH nanopillars are developed to boost the reaction kinetics of anodic GOR and cathodic hydrogen evolution, respectively. Meanwhile, excluding the explosion risk of mixed H_2_/O_2_, a cheap organic membrane is used to replace the expensive anion exchange membrane in the electrolyzer. Impressively, the electrolyzer delivers a remarkable reduction of operation voltage by 280 mV, and exhibits good long-term stability. This work provides a new paradigm of hydrogen production with low cost and good feasibility.

**Supplementary Information:**

The online version contains supplementary material available at 10.1007/s40820-023-01150-1.

## Highlights

- Two types of vertical 3D nanostructures were successfully fabricated using simple hydrothermal and heat treatment processes for hydrogen evolution reaction and glycerol oxidation reaction (GOR).
- Hydrogen production at a lower potential was achieved by replacing oxygen evolution reaction with GOR, reducing the device potential by approximately 300 mV. Additionally, organic membranes were used as separators, avoiding the use of expensive anion exchange membranes.

**Supplementary Information:**

The online version contains supplementary material available at 10.1007/s40820-023-01150-1.

## Graphical abstract

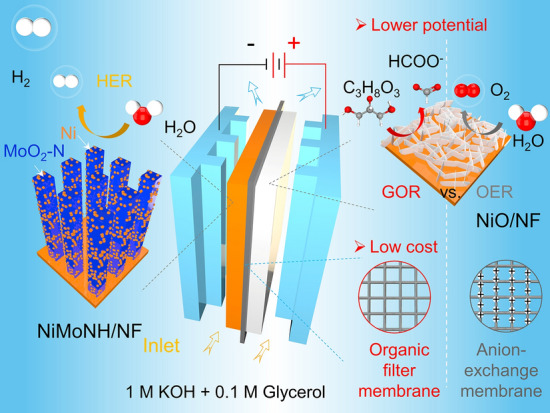

## Introduction

Water electrolysis technology is promising to convert clean electricity generated by intermittent wind and solar energy into storable green hydrogen energy, which is one of the important paths to achieve carbon neutrality [1–4]. The electrolytic water process usually consists of two half-reactions, the anodic oxygen evolution reaction (OER) and the cathodic hydrogen evolution reaction (HER). However, the anodic four-electron OER with slow kinetics and high overpotential is the key limitation of the water splitting [5–7]. A great deal of studies in the past have focused on the design and development of advanced OER electrocatalysts to reduce the power consumption and cost of hydrogen production from electrolytic water. However, the anodic product oxygen (O_2_) is usually a worthless and energy-consuming by-product accompanying hydrogen production from electrolytic water. In addition, the generated gas products may form hazardous H_2_/O_2_ mixture, which require additionally costly proton exchange membrane (PEM, $4.2 kg^−1^) or anion-exchange membrane (AEM, $3.7 kg^−1^) [8] to separate the cathode and anode chambers. Furthermore, the generated reactive oxygen species (ROS) will shorten the lifetime of the membrane [9, 10]. In a word, the high energy consumption and the safety issues had limited the development of electrolytic water.

Recently, a number of thermodynamically and economically favorable oxidation reactions have been widely explored to replace the sluggish OER, which were coupled with the HER for H_2_ production [6, 7, 11]. Among these alternative reactions, one type is the oxidation of sacrificial agents, such as hydrazine oxidation reaction (HzOR, N_2_H_4_ + 4OH^−^ → N_2_ + 4H_2_O + 4e^−^, − 0.33 V vs. RHE) [12, 13], urea oxidation reaction (UOR, CO(NH_2_)_2_ + 6OH^−^  → N_2_ + CO_2_ + 5H_2_O + 6e^−^, 0.37 V vs. RHE) [14, 15], whose products are N_2_ and other safe gases. Another one is the organic upgrading reaction, which can significantly reduce the anode potential and obtain high value-added chemicals at the same time, such as methanol oxidation reaction (MOR, CH_3_OH + 5OH^−^  → HCOO^−^  + 4H_2_O + 4e^−^) [16–19], glycerol oxidation reaction (GOR, C_3_H_8_O_3_ + 8OH^−^ → 3HCOOH + 5H_2_O + 8e^−^, 0.69 V vs. SHE) [20, 21], 5-hydroxymethyl furfural (HMF) oxidation [22, 23], aldehydes oxidation [8, 24], glucose oxidation [25, 26]. It is noted that coupling HER with the oxidation reaction of organic molecules can achieve the acquisition of H_2_ and high value-added organic molecules at low potentials. Glycerol is a low-value byproduct of biodiesel production [27], while its oxidation product formate (HCOO^−^) or formic acid (HCOOH) is widely used in industrial production [20]. Unfortunately, it is still highly in demand on low-cost and highly active catalysts for glycerol oxidation.

**Scheme 1:**
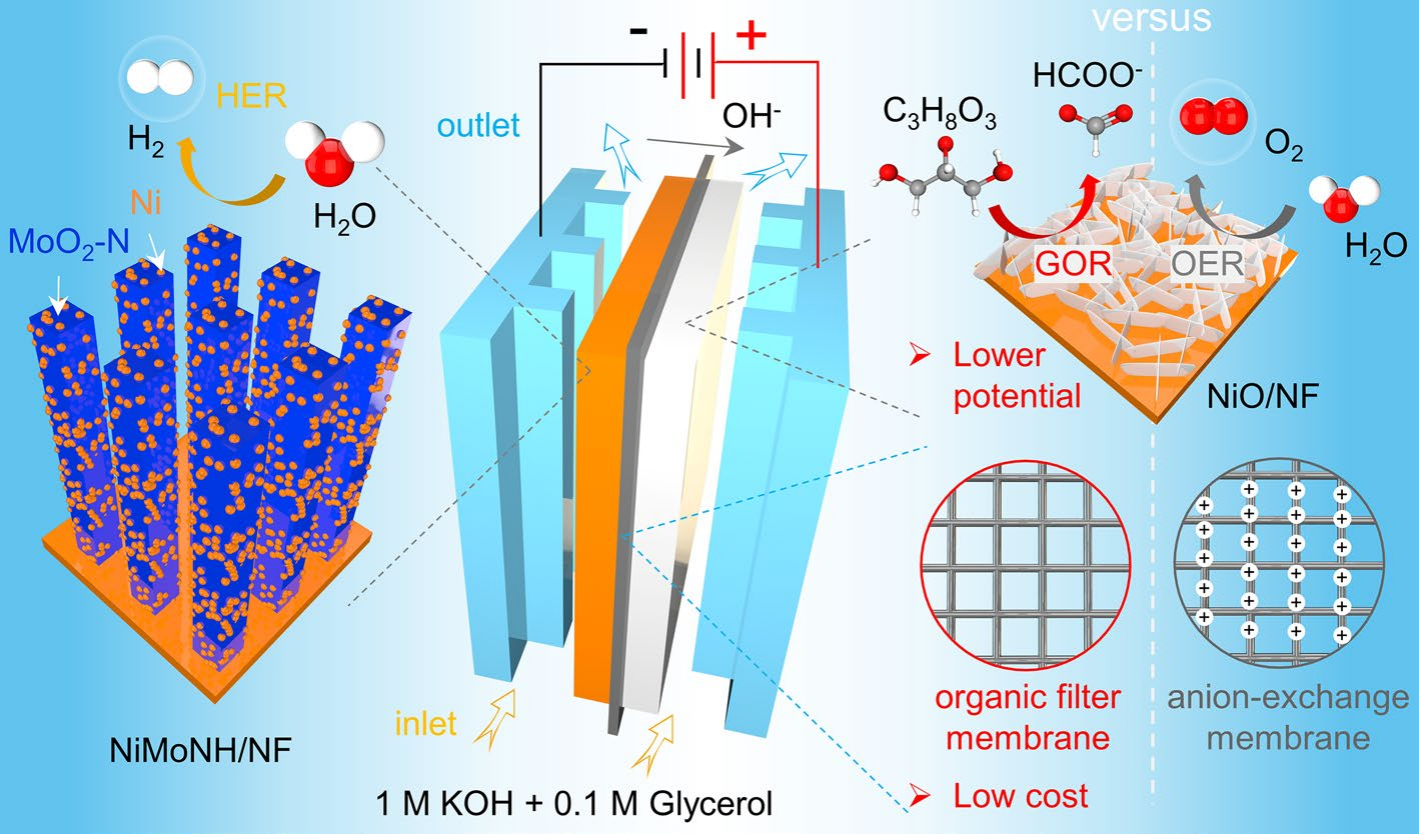
Design of the hybrid water electrolyser

In this work, we develop a hybrid water electrolysis flow cell for hydrogen production coupled to glycerol oxidation based on a nickel-based catalyst and using inexpensive organic membranes (Scheme 1). Specifically, for the anodic glycerol oxidation reaction (GOR), we used nickel oxide (NiO) nanosheet arrays as an electrocatalyst, which exhibited better activity than nickel hydroxide (Ni(OH)_2_). The addition of glycerol molecules significantly reduces the anodic potential compared to the conventional OER. At the cathode, we used NiMoNH nanopillar arrays as a HER electrocatalyst, which were obtained by annealing NiMoO in ammonia (NH_3_) and argon–hydrogen (Ar/H_2_) mixture gas. The addition of glycerol molecules remarkably decreases the voltage of electrolyze, which is beneficial for the reduction of energy consumption. Moreover, the alkaline anion exchange membrane can be replaced with an inexpensive organic filter membrane, without degradation of electrochemical performance. This study opens up a new point of view for the future exploration of practical hybrid water electrolyzer.

## Experimental Section

### Preparation of Catalysts

#### ***Synthesis of Ni(OH)***_***2***_

A piece of nickel foam (NF) (3 cm × 6 cm) was first cleaned by sonicating in 0.1 M HCl, deionized water, and ethanol for 15 min each. Then, 1.45 g nickel (II) nitrate hexahydrate (Ni(NO_3_)_2_·6H_2_O) and 1.4 g hexamethylenetetramine (HMT) were dissolved in 70 mL deionized water and stirred for 30 min. This solution was then poured into a Teflon-lined stainless steel autoclave, and then the cleaning NF was immersed in it before being heated to 120 °C for 6 h. After cooling down to room temperature, the resulting Ni(OH)_2_ nanosheets on the NF were rinsed with deionized water and left to dry naturally.

#### Synthesis of NiO

The as-grown Ni(OH)_2_ precursor was heated in a quartz tube furnace at 300 °C for 3 h under air to convert into NiO.

#### Synthesis of NiMoO

1.4 g Ni(NO_3_)_2_·6H_2_O and 1.48 g ammonium molybdate tetrahydrate ((NH_4_)_6_Mo_7_O_24_·4H_2_O) were dissolved in 80 mL deionized water and stirred for 30 min. Then, this solution was transferred to a Teflon-lined stainless steel autoclave, and then immersed the NF into the solution and heated to 150 °C for 6 h. After cooling down to room temperature, the obtained NiMoO supported on NF were washed with deionized water and then dried naturally.

#### Synthesis of NiMoNH

The as-grown NiMoO precursor on the NF was annealed in a quartz tube furnace at 400 °C for 2 h in an NH_3_ atmosphere to turn it into NiMoN. The NiMoN was then heated in a quartz tube furnace at 500 °C for 2 h in an Ar/H_2_ atmosphere to form NiMoNH.

### Characterization

#### Materials Characterization

A field emission scanning electron microscope (FEI Magellan 400L XHR) was used for obtaining the scanning electron microscopy (SEM) images. A Titan G2 60–300 Cs-corrected transmission electron microscopy (TEM) was used for TEM, high-resolution TEM (HRTEM), high angle annular dark-field scanning TEM (HADDF-STEM), and energy-dispersive X-ray spectroscopy (EDS) mapping. X-ray diffraction (XRD) measurements were performed on a Bruker D8 ADVANCE X-ray diffraction diffractometer. A Thermo ESCALAB250xi electron spectrometer with an Al Kα source (1486.6 eV) as radiation source was used for X-ray photoelectron spectroscopy (XPS) measurements. A Bruker A300 spectrometer was used for acquiring the Electron Paramagnetic Resonance (EPR) spectra.

#### Electrochemical Characterization

We used a CHI 760E electrochemical workstation to perform electrochemical measurements in an H-type three-electrode cell (H cell). A graphite rod electrode and a Hg/HgO were used as the counter electrode and reference electrode, respectively. The working electrode (1 cm × 1 cm) was using the as-prepared self-supporting electrodes. Fumasep FAB-PK-130 was used as the anion exchange membrane (AEM). All the potentials versus Hg/HgO were converted to the values versus the reversible hydrogen electrode (RHE) using the equation: E vs. RHE = E versus Hg/HgO + 0.924 V.

We prepared a membrane electrode assembly (MEA) for testing the electrochemical performance in a flow cell (Gaossunion Co., Ltd., Tianjin, China). The MEA was made by sandwiching NiO and NiMoNH electrode between either a commercial membrane (Fumasep FAB-PK-130) or an organic membrane. The MEA was then placed within a custom-designed electrolyzer where 1 M KOH and 0.1 M glycerol were circulated through cathode and anode as the electrolyte.

## Results and Discussion

### Characterization of NiO and Ni(OH)_2_

**Fig. 1:**
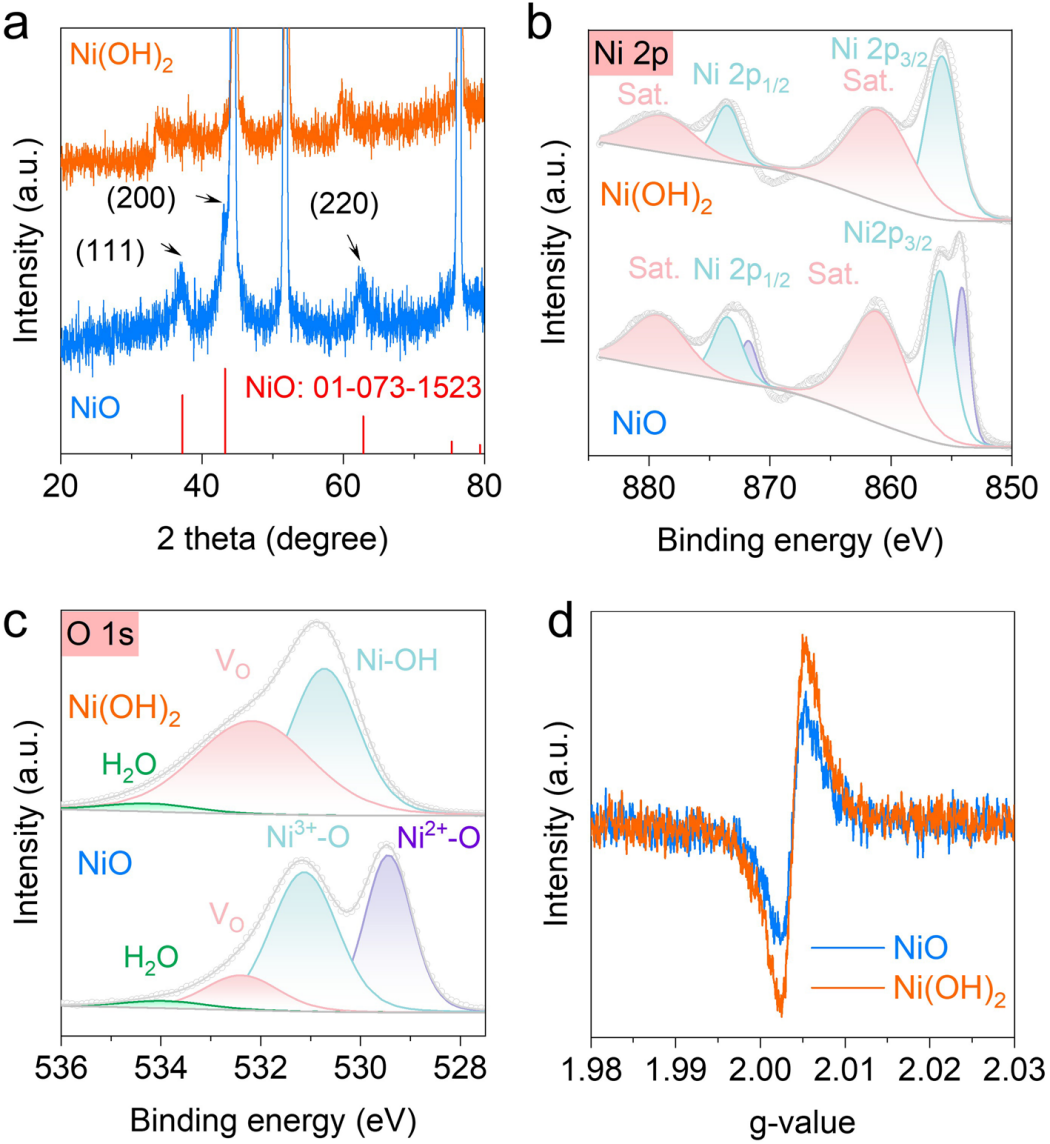
Structure characterization of Ni(OH)_2_ and NiO. **a** XRD patterns of the Ni(OH)_2_ and NiO catalysts. High-resolution XPS spectra of **b** Ni 2*p* and **c** O 1*s* for Ni(OH)_2_ and NiO catalysts. **d** EPR spectra of the Ni(OH)_2_ and NiO catalysts

Figure S1 shows the synthesis of NiO catalyst. A commercially available nickel foam (NF) was used as the conductive substrate. Firstly, the nickel hydroxide (Ni(OH)_2_) precursor grew on the NF through a hydrothermal reaction of nitrate hexahydrate and hexamethylenetetramine in a mixed solution [28]. Then, the Ni(OH)_2_ was heated at 300 °C for 3 h under air atmosphere to form nickel oxide (NiO). The XRD pattern (Fig. 1a) shows that the diffraction peaks are indexed to NiO.

The chemistry state of the Ni(OH)_2_ and NiO were investigated by X-ray photoelectron spectroscopy (XPS). The high-resolution Ni 2*p* XPS spectra is shown in Fig. 1b. The Ni 2*p* show two major peaks located at 873 and 855 eV, which can be attributed to the Ni 2*p*_1/2_ and Ni 2*p*_3/2_, respectively. The Ni 2*p*_3/2_ peak of NiO is deconvoluted into two prominent peaks located at 854.1 and 856.0 eV attributed to Ni^2+^ and Ni^3+^ of NiO, respectively [29]. Figure 1c shows the O 1*s* spectra of Ni(OH)_2_ and NiO. The peak with the highest intensity at 529.5 eV is assigned to Ni^2+^–O in NiO. The peak at 531.1 eV is related to oxygen-containing species, which is assigned to Ni^3+^–O [29]. The weak peaks at 532.2 and 534 eV belong to oxygen vacancy (V_O_) and adsorbed H_2_O, respectively [30, 31]. The area of V_O_ for Ni(OH)_2_ is much higher than that of NiO. The electron paramagnetic resonance (EPR) spectrum (Fig. 1d) further confirms the existence of V_O_ in Ni(OH)_2_ and NiO. Ni(OH)_2_ shows a strong EPR signal at around g = 2.003, indicating rich V_O_ in Ni(OH)_2_ [32], which agrees with the result of XPS. In addition, the Brunauer–Emmett–Teller (BET) specific surface area of NiO and Ni(OH)_2_ was tested. As shown in Fig. S2, NiO has a higher specific surface area (5.67 m^2^ g^−1^) than nickel Ni(OH)_2_ (3.49 m^2^ g^−1^).

**Fig. 2:**
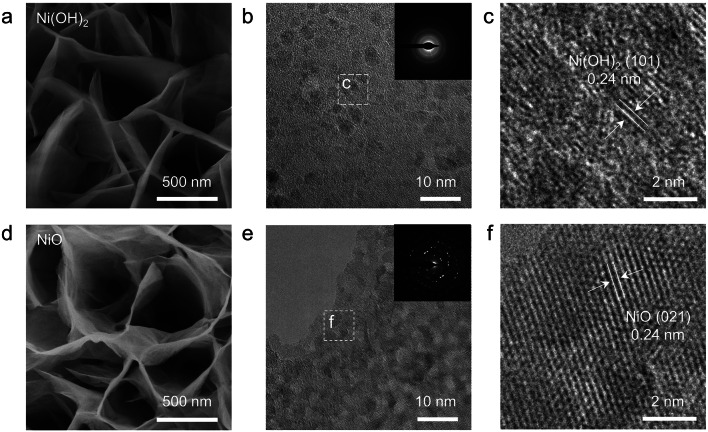
Morphology characterization of the Ni(OH)_2_ and NiO catalysts. **a** SEM, **b** TEM and **c** HRTEM images of Ni(OH)_2_. **d** SEM, **e** TEM and **f** HRTEM images of NiO

SEM and TEM was used to study the morphology of Ni(OH)_2_ and NiO. Figures 2a and S1b–c show that the original Ni(OH)_2_ possesses a well-defined 2D nanosheet morphology. After the annealing, the 2D nanosheet morphology is still retained for the NiO samples (Figs. 2d and S1d–e). Figure 2b displays the TEM image of Ni(OH)_2_. The inset of Fig. 2b shows the selected area electron diffraction (SAED) pattern of Ni(OH)_2_ nanosheet. The pattern shows diffused rings, which means the as-prepared Ni(OH)_2_ nanosheet has poor crystallinity. Thera are no typical lattice fringes from the high-resolution TEM (HRTEM) image of Ni(OH)_2_ sample (Fig. 1c), which suggests that the as-prepared Ni(OH)_2_ nanosheets are amorphous. Figure S3 shows the high-angle annular dark-field scanning TEM (HAADF-STEM) image and the corresponding EDS elemental mappings of Ni(OH)_2_. Ni and O elements were uniformly distributed throughout the nanosheet. The TEM image (Fig. 2e) and SAED pattern (inset of Fig. 2e) of the NiO shows good crystallinity. The HRTEM image of NiO (Fig. 2f) shows the lattice fringes of (021) planes. Figure S4 shows the HAADF-STEM image and the corresponding EDS elemental mappings of NiO.

### Electrocatalytic Performances of Ni(OH)_2_ and NiO for GOR

**Fig. 3:**
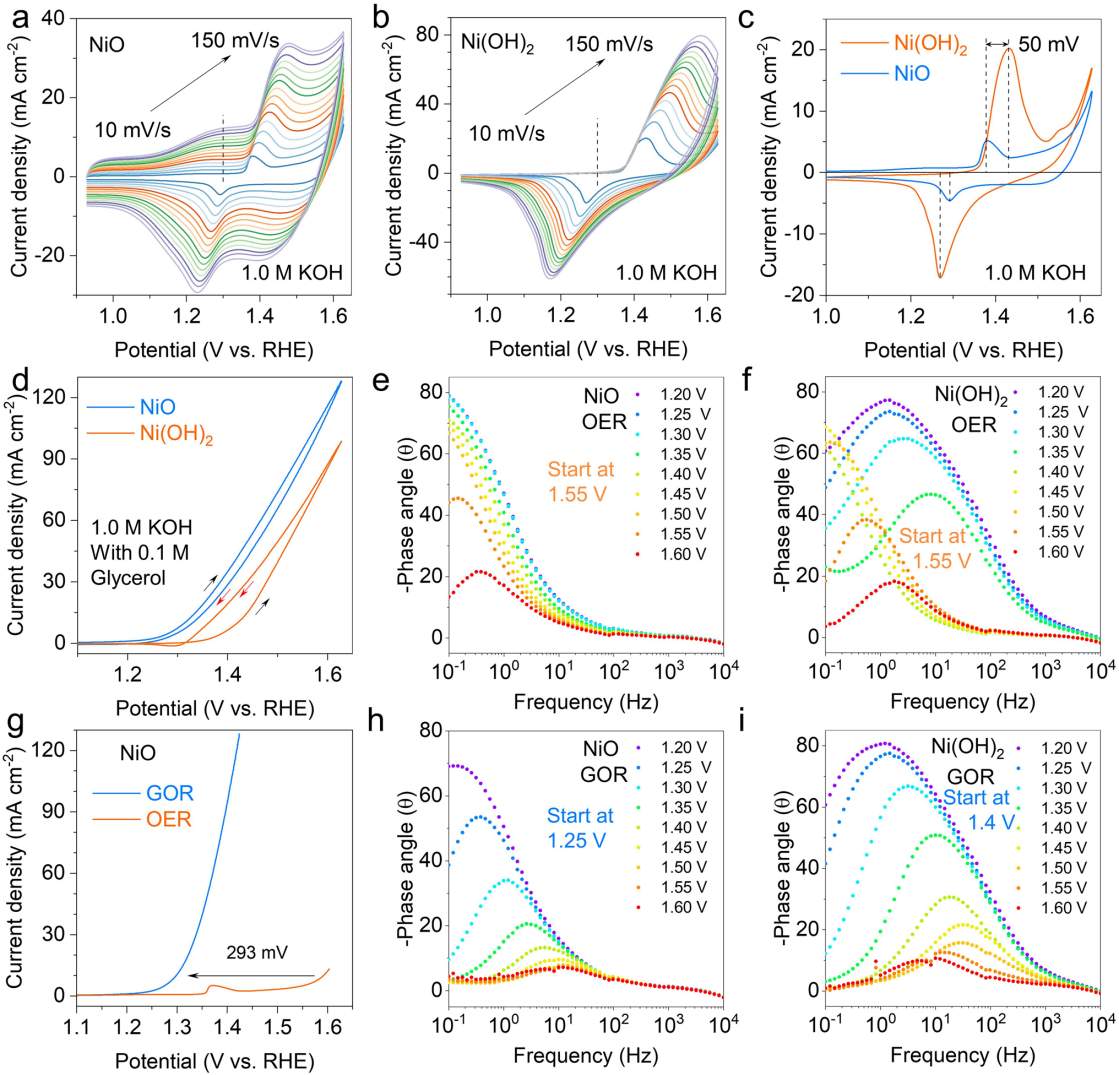
Electrocatalytic performances of Ni(OH)_2_ and NiO for GOR. Cyclic voltammograms of **a** NiO and **b** Ni(OH)_2_ electrocatalysts in 1 M KOH at different sweep rates. **c** Comparison of cyclic voltammograms of NiO and Ni(OH)_2_ in 1.0 M KOH at 10 mV s^−1^. **d** CV cures of NiO and Ni(OH)_2_ for GOR. **g** LSV cures of NiO for GOR and OER. Bode plots for the **e** NiO and **f** Ni(OH)_2_ in 1 M KOH. Bode plots for the **h** NiO and **i** Ni(OH)_2_ in 1 M KOH with 0.1 M glycerol

The electrochemical performance of NiO and Ni(OH)_2_ was tested in 1.0 M KOH electrolyte with and without 0.1 M glycerol, respectively. Firstly, a typical cyclic voltammetry (CV) cycling was carried out in 1 M KOH medium. Figure 3a, b shows the CV cures of NiO and Ni(OH)_2_ electrodes at various scan rates, respectively. When the scan rate goes up, the oxidation peak moves to more positive potential, and the reduction peak moves to a more negative potential. Generally, the proton diffusion rate determines the speed of the oxidation of Ni(OH)_2_ to NiOOH [33]. The peak current densities were plotted against the square roots of the scan rates in Fig. S5 and a linear relationship was found, Ni(OH)_2_ shows higher proton diffusion coefficient than NiO. In addition, it is found that the current density of NiO between 1.0 and 1.35 V increases with the increase of scan rate (Fig. 3a), but this phenomenon is not observed in Ni(OH)_2_ (Fig. 3b). Which means that the NiO surface has better adsorption for OH species [21]. Figure 3c shows the CV curves of NiO and Ni(OH)_2_ at 5 mV s^−1^, where the Ni oxidation peak potential for NiO is lower than that for Ni(OH)_2_, indicating that NiO is easier to be oxidized.

The electrocatalytic performance of NiO and Ni(OH)_2_ towards the GOR was tested in an electrolyte of 1 M KOH containing 0.1 M glycerol. From the CV cures in Fig. 3d, NiO has the lower onset potential than Ni(OH)_2_, showing better electrochemical performance. A CV method was used to measure the electrochemical double-layer capacitance (*C*_dl_) of the catalysts and evaluate their electrochemically surface area (ECSA) from *C*_dl_ (Fig. S6) in order to better understand the improvement of the GOR activity. The results show that NiO has a considerably larger *C*_dl_ (12.3 mF cm^−2^) than Ni(OH)_2_ (0.79 mF cm^−2^). From the linear sweep voltammetry (LSV) curve with IR correction (Fig. 3g), it can be seen that the addition of glycerol to the KOH electrolyte significantly reduces the reaction potential at the anode (293 mV). This suggests that the operating potential during hydrogen production from electrolytic water is expected to be reduced by replacing the OER with GOR. Inspire by this, OER can be replaced by GOR to reduce the potential of hydrogen production by water electrolysis, and at the same time, high value-added formate can be produced at the anode [20]. Compared with other reported transition metal catalysts, NiO exhibits better GOR performance (Table S1). We also compared the electrocatalytic oxidation performance of NiO and Ni(OH)_2_ in 1 M KOH containing 0.1 M other alcohols (including ethylene glycol, ethanol, methanol), as shown in Fig. S7. It can be seen that for the ethylene glycol oxidation reaction (EGOR, Fig. S7a), the oxidation potential of NiO is lower than that of Ni(OH)_2_; in the ethanol oxidation reaction (EtOR, Fig. S7b), there is no significant difference; in the methanol oxidation reaction (MOR, Fig. S7e), Ni(OH)_2_ is better than NiO. Overall, NiO seems to have a differential ability to oxidize different alcohols (Fig. S7c).

The difference of electrochemical activity between NiO and Ni(OH)_2_ were further studied by the in-situ EIS during the OER and GOR (Figs. 3e, f, h, i and S8). From the Bode phase plots, Ni(OH)_2_ has a peak in the low-frequency (10^0^–10^1^ Hz) region between 1.2 and 1.35 V during the OER (Fig. 3f), which may be related to the formation of oxide species on the electrode surface [22]. But this phenomenon is not observed in NiO (Fig. 3e). When the potential is increased to 1.55 V, new peaks (about 10^–1^–10^0^ Hz) appear in both NiO and Ni(OH)_2_, which may be associated with the start of the OER. After adding 0.1 M glycerol into the KOH, a peak conversion was examined at the potential of 1.25 V for NiO (Fig. 3h) and 1.4 V and Ni(OH)_2_ (Fig. 3i), respectively, for GOR. Furthermore, in the Nyquist plots (Fig. S8), the charge-transfer resistance of NiO is much smaller than that of Ni(OH)_2_. The EIS analysis shows that the lower charge-transfer resistance of NiO accelerates the kinetics of GOR.

Further, a chronopotentiometric measurement was used to test the stability of NiO for GOR (Fig. S9). As the oxidation reaction continues, glycerol is gradually consumed and the potential decreases. The XPS of NiO and Ni(OH)_2_ after the GOR was also tested, as shown in the Figs. S10 and S11. The oxidation peak area of Ni in NiO increases after GOR (Fig. S10a). In addition, a new C=O peak is observed in the C 1* s* spectrum (Fig. S10c), indicating the existence of residual formate. From the SEM image in Fig. S12, it can be seen that after GOR, NiO still maintains its 3D nanosheet stacked structure without significant changes. And there is also no significant change in the XRD pattern (Fig. S13).

### Characterization of the NiMoNH HER Catalysts

**Fig. 4:**
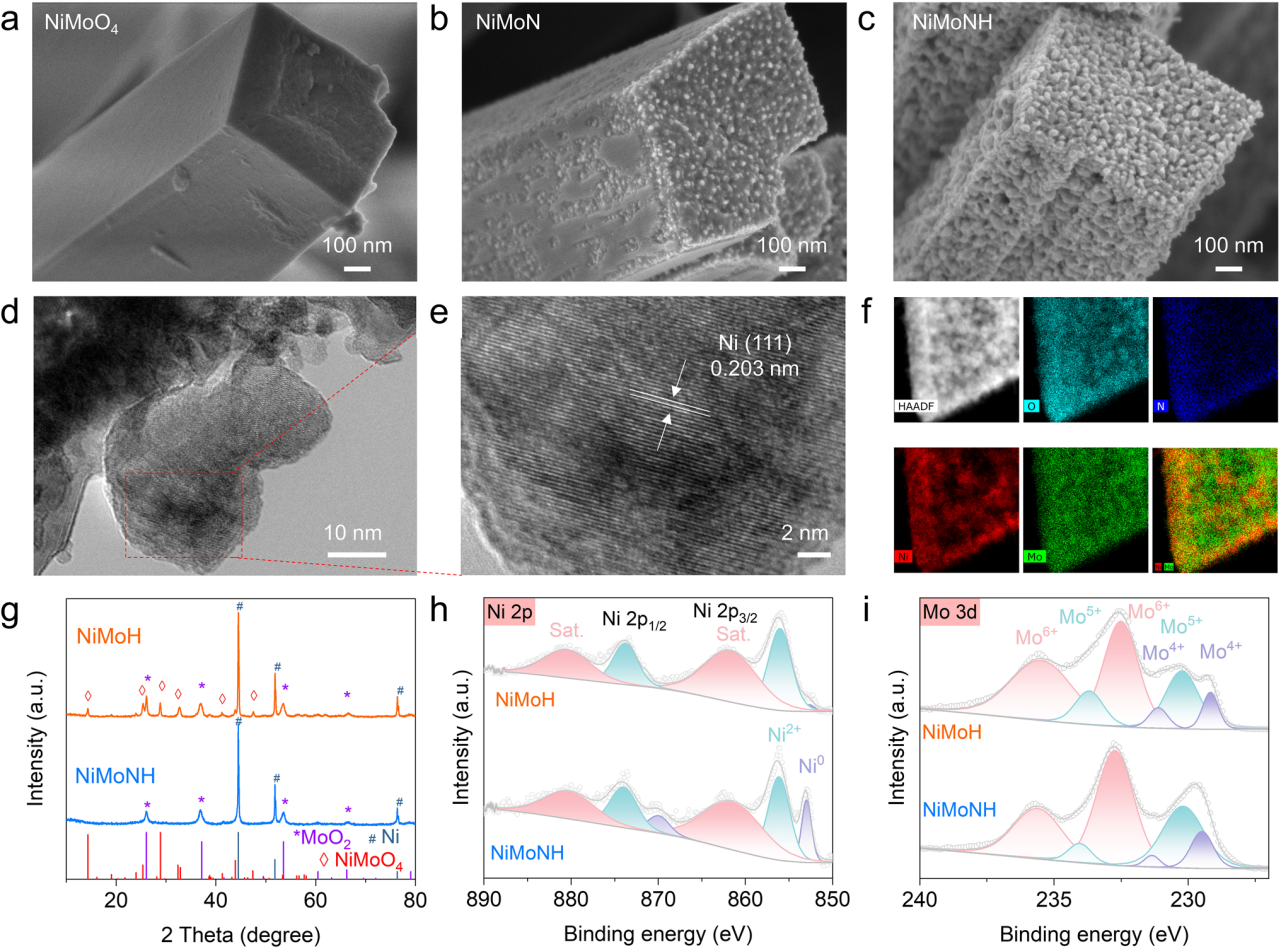
Characterization of the NiMoNH HER catalysts. SEM images of **a** NiMoO, **b** NiMoN and **c** NiMoNH. **d** TEM image, **e** HRTEM image, **f** HADDF-STEM and EDS mapping images of NiMoNH. **g** XRD pattern, **h** Ni 2*p* XPS spectra and **i** Mo 3*p* XPS spectra of NiMoH and NiMoNH

To couple the anodic GOR, a nickel-molybdenum-based catalyst for cathodic HER is also developed, whic is a very promising non-precious metal electrocatalyst. Firstly, NiMoO nanoarrays was grown on NF matrix by a hydrothermal method, and then highly active hydrogen evolution catalyst (denoted as NiMoNH) was obtained by annealing in NH_3_ and Ar/H_2_ atmosphere (Fig. S14). SEM images (Figs. 4a–c and S14) show the morphology change from NiMoO to NiMoNH. In Fig. 4a and S14b–c, it is noted that NiMoO display nanopillar array and relatively smooth surface. After annealing in NH_3_ atmosphere, some nanoparticles appeared on the surface and became rough (Figs. 4b and S14e–f). Finally, after further calcination in an Ar/H_2_ atmosphere, the surface of NiMoNH nanopillars are covered with nanoparticles and become extremely rough (Figs. 4c and S14h–i). The unique 3D nanoarray structure will facilitate the release of hydrogen [34, 35]. In addition, the sample annealed directly in argon–hydrogen atmosphere was also synthesized as a control sample (note as NiMoH). The particle size on its surface is slightly larger than that of NiMoNH (Fig. S15).

TEM images further reveal the microstructure of NiMoNH (Figs. 4d and S16). From the HRTEM image (Fig. 4e), the NiMoNH nanoparticles show the lattice fringes of 0.203 nm, which is related to the (111) plane of Ni. Aggregation of Ni particles is also verified in the HADDF-STEM image with the corresponding EDS mappings (Fig. 4f). XRD pattern in Fig. 4g reveals that NiMoNH consists of MoO_2_ and Ni phases. While the NiMoO_4_ phase can still be found in NiMoH, which indicates incomplete conversion of NiMoO_4_.

The chemical states of NiMoNH and NiMoH were further analyzed by XPS. As shown in Fig. 4h, the high-resolution Ni 2*p*_3/2_ XPS spectra of NiMoNH exhibit a clear intensity increase at ~ 853 eV, which proves the existence of metal Ni (0). The high-resolution Mo 3*d* signal of NiMONH and NiMoH (Fig. 4i) were fitted into three typical components at 229.2, 230.4, and 232.2 eV, corresponding to Mo^4+^, Mo^5+^, and Mo^6+^, respectively [36]. In the N 1* s* XPS spectrum of NiMoNH (Fig. S17), there is a peak at 397.4 eV that corresponds to the N species in metal nitrides, and another peak at 399.6 eV that is due to the incomplete reaction of NH_3_ [37].

### HER Performance of NiMoNH

**Fig. 5:**
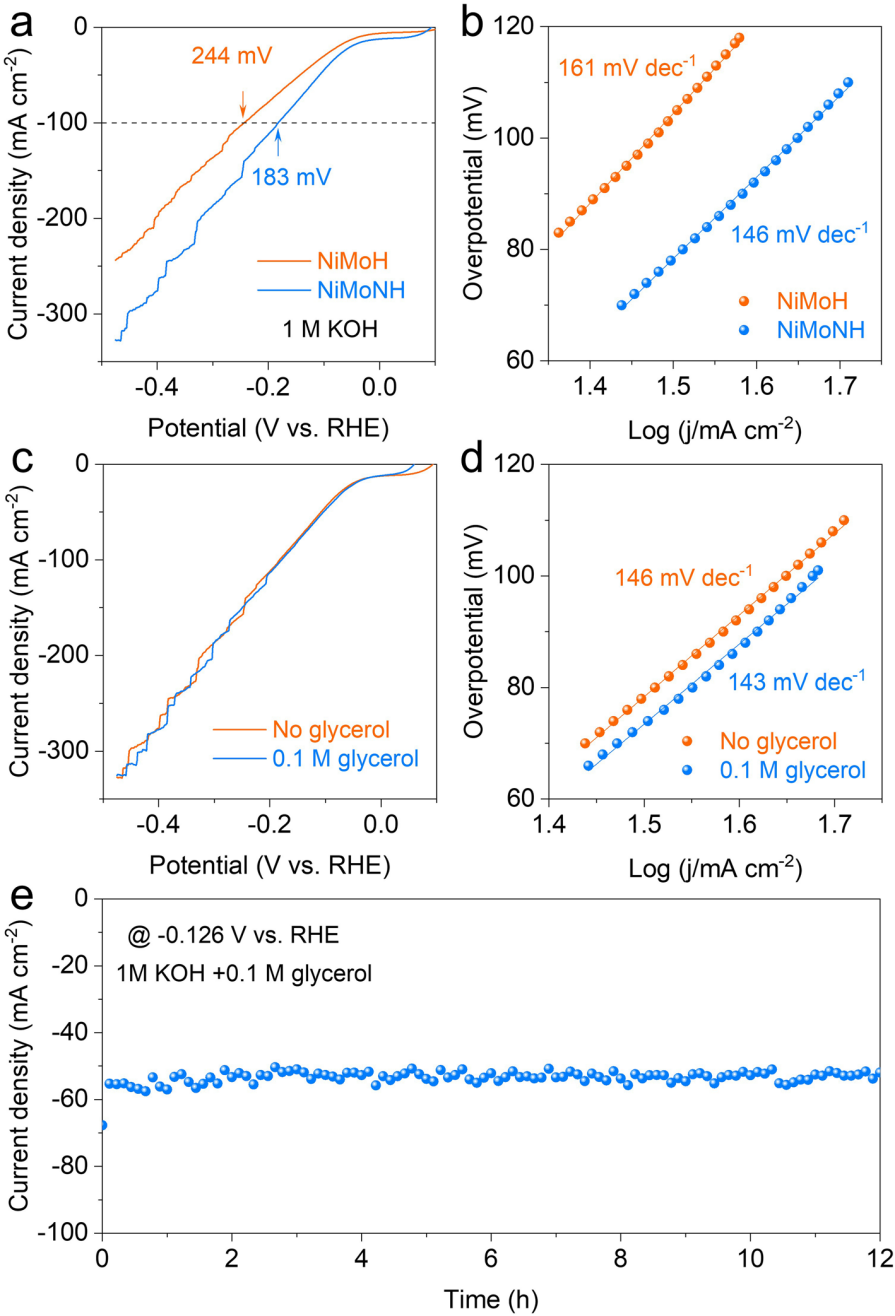
HER performance of NiMoNH. **a** LSV curves and **b** the corresponding Tafel plots of NiMoNH and NiMoH in 1.0 M KOH. **c** LSV curves and **d** the corresponding Tafel plots of NiMoNH in 1.0 M KOH with and without 0.1 M glycerol. **e** Chronoamperometry curves (i–t) recorded on NiMoNH for HER at − 0.126 V vs. RHE

The HER performance of NiMoNH and NiMoH on Ni foam was tested in 1 M KOH. In Fig. 5a, the LSV curves show that NiMoNH exhibited the better catalytic activity than NiMoH. The overpotential requirements at current densities of 100 mA cm^−2^ were ~ 183 and 224 mV for the NiMoNH and NiMoH, respectively. NiMoNH and NiMoH exhibit the Tafel slope (Fig. 5b) of 146 and 161 mV dec^−1^, respectively. The HER performance of NiMoNH catalyst in 1 M KOH containing 0.1 M glycerol was further examined. Excitingly, the addition of glycerol has no obvious effect on the HER performance of NiMoNH (Fig. 5c), which is extremely important for the subsequent development of GOR-assisted hydrogen production. As shown in Fig. 5d, NiMoNH shows the similar Tafel slopes in different electrolyte medium, which means that it has similar catalytic kinetics. Compared with other NiMo-based catalysts (Table S2), NiMoNH has similar HER performance. In addition, the stability of NiMoNH was also tested as shown in Fig. 5e. NiMoNH can perform stably at − 0.126 V vs. RHE for 12 h and is potential to be used as a cathode for electrolyzer. SEM images (Fig. S18) and XRD pattern (Fig. S19) of NiMoNH after stability testing showed no significant changes in the morphology and structure of the material, with good stability.

### Performance of Hybrid Electrolyzer

**Fig. 6:**
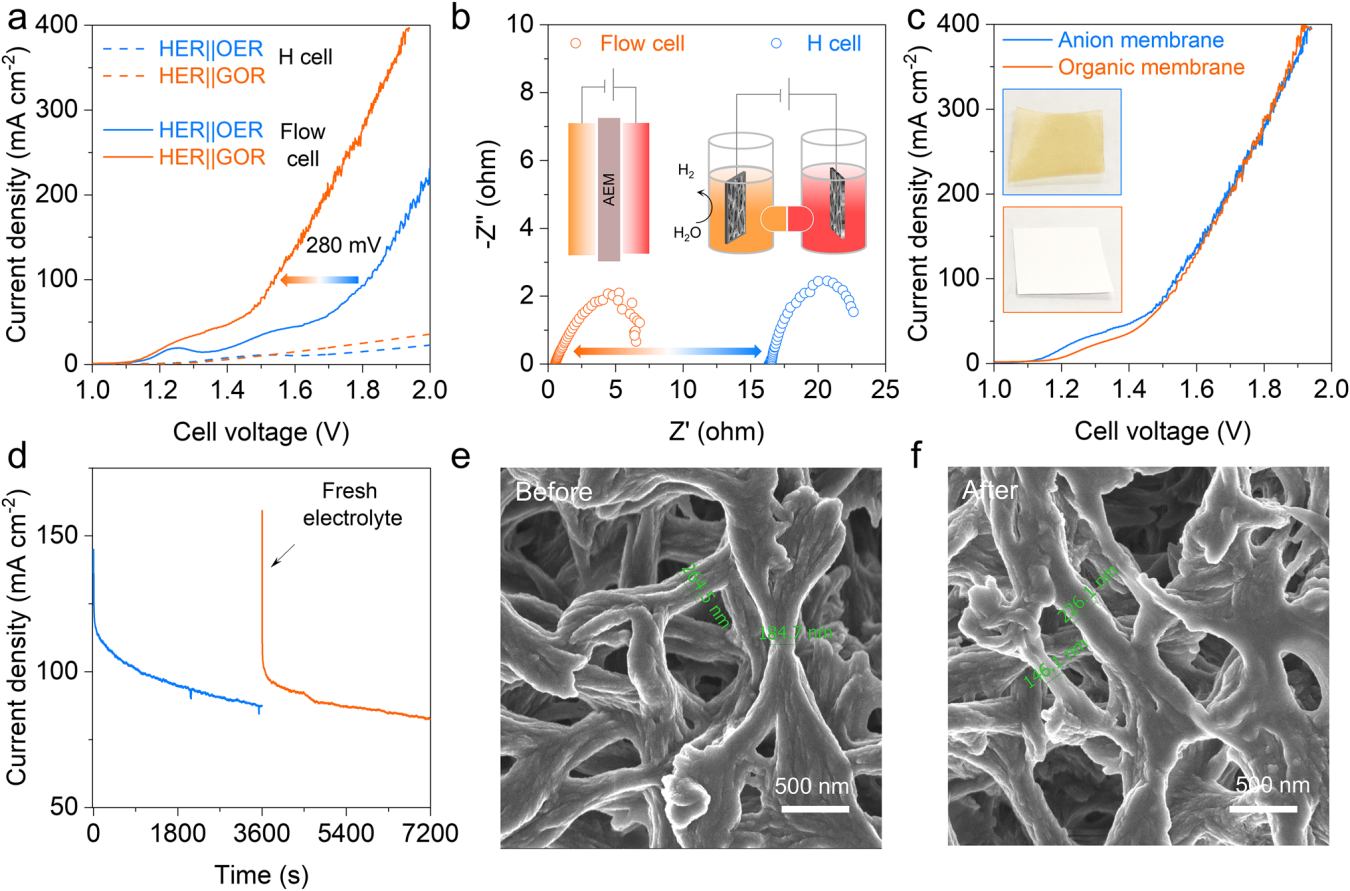
Performance of hybrid electrolyzer (using NiO/NF as anodic electrocatalyst and NiMoNH/NF as cathodic electrocatalyst). **a** Polarization curve of the hybrid electrolyzer. **b** Nyquist plots from the EIS measurements for MEA cell and H cell. **c** Polarization curve of the hybrid electrolyzer with alkaline anion membrane and organic membrane. **d** Chronoamperometry curve of the hybrid electrolyzer using organic membrane at 1.6 V. SEM images of organic membrane **e** before and **f** after long-term testing

Considering that the as-synthesized NiO and NiMoNH have excellent GOR and HER properties, respectively. A H-type electrolyzer with membrane was used to test their hybrid electrolysis performance. Figure 6a shows that the LSV curves of the electrolyzer with and without glycerol. The anion exchange membrane (AEM) flow cell represented by the solid line exhibits a higher current density than the H-type cell (dash line). The GOR-assisted hydrogen production (HER||GOR) only requires a cell voltage of 1.54 V at a current density of 100 mA cm^−2^, which is remarkably reduced by 280 mV comparted to the overall water splitting (HER||OER). The advantage of the flow cell was further confirmed by electrochemical impedance spectroscopy (EIS) analysis (Fig. 6b). It can be seen from the Nyquist plots that the contact resistance of the flow cell is much smaller than that of H cell.

Considering that the anode OER is replaced by GOR, this process has no oxygen generation and cannot induce explosive H_2_/O_2_ mixtures. Therefore, a cheaper organic filter membrane (Nylon) was used to replace the expensive AEM (inset of Fig. 6c). The NiMoNH cathode, organic membrane and NiO anode were assembled in the flow cell. The pores of the organic membrane allow the free transport of K^+^ and OH^−^, while the physical isolation of cathode and anode is realized. As shown in Fig. 6c, the replacement of the membrane has not induced degradation of the electrochemical performance. Compared to other small molecule oxidation assisted hydrogen production devices (Table S3), the electrolyzer potential assembled in this work is lower. Moreover, the assembled flow cell also exhibits good stability (Fig. 6d), where the drop in current is associated with the depletion of glycerol molecule. The morphology of the organic member before and after stability test of the flow cell were investigated by SEM. As shown in Fig. 6d, the organic membrane has abundant pores for ion transport and mass transfer. No obvious change is observed after the stability test (Fig. 6f), which indicates that the organic membrane is feasible to be used in HER||GOR system at low cost for long-term operation.

## Conclusions

In summary, vertical 3D NiO nanoflakes and NiMoNH nanopillars have been successfully synthesized to use as electrocatalysts for the anodic GOR and cathodic HER, respectively. The replacement of OER with GOR together with the highly active NiO nanoflakes remarkably reduces the operation voltage of electrolyzer for hydrogen evolution. Furthermore, cheaper organic membrane instead of an anion exchange membrane is employed as separator to lower the whole cost of electrolyzer. The as-assembled electrolyzer exhibits good HER performance and long-term stability. This work opens a new avenue for the practical applications in the future hydrogen economy.

### Supplementary Information

Below is the link to the electronic supplementary material.

Supplementary file1 (DOCX 8857 KB)
