# Supplementary material for: Vertical 3D Nanostructures Boost Efficient Hydrogen Production Coupled with Glycerol Oxidation Under Alkaline Conditions
Source: Nanomicro Lett. 2023 Jul 29;15:189. doi: 10.1007/s40820-023-01150-1 (PMC10387032; doi:10.1007/s40820-023-01150-1)
Supplement: Supplementary file 1 — Supplementary file1 (DOCX 8857 KB) [file 40820_2023_1150_MOESM1_ESM.docx]

**Vertical 3D nanostructures boost efficient hydrogen production coupled with glycerol oxidation under alkaline** **conditions**

Shanlin Li^1, 2^, Danmin Liu^1, *^, Guowei Wang^1^, Peijie Ma^1^, Xunlu Wang^2^, Jiacheng Wang^2, 4, 5, *^, and Ruguang Ma^3,*^

^1^ Key Laboratory of Advanced Functional Materials, Ministry of Education, Faculty of Materials and Manufacturing, Beijing University of Technology, Beijing 100124, China.

^2^ The State Key Laboratory of High Performance Ceramics and Superfine Microstructure, Shanghai Institute of Ceramics, Chinese Academy of Sciences, Shanghai 200050, China.

^3^ School of Materials Science and Engineering, Suzhou University of Science and Technology, 99 Xuefu Road, Suzhou 215011, China.

^4^ School of Materials Science and Engineering, Taizhou University, Taizhou 318000, China.

^5^ Hebei Provincial Key Laboratory of Inorganic Nonmetallic Materials, College of Materials Science and Engineering, North China University of Science and Technology, Tanshang 063210, China.

*Corresponding author. E-mail: dmliu@bjut.edu.cn; jiacheng.wang@mail.sic.ac.cn; ruguangma@usts.edu.cn


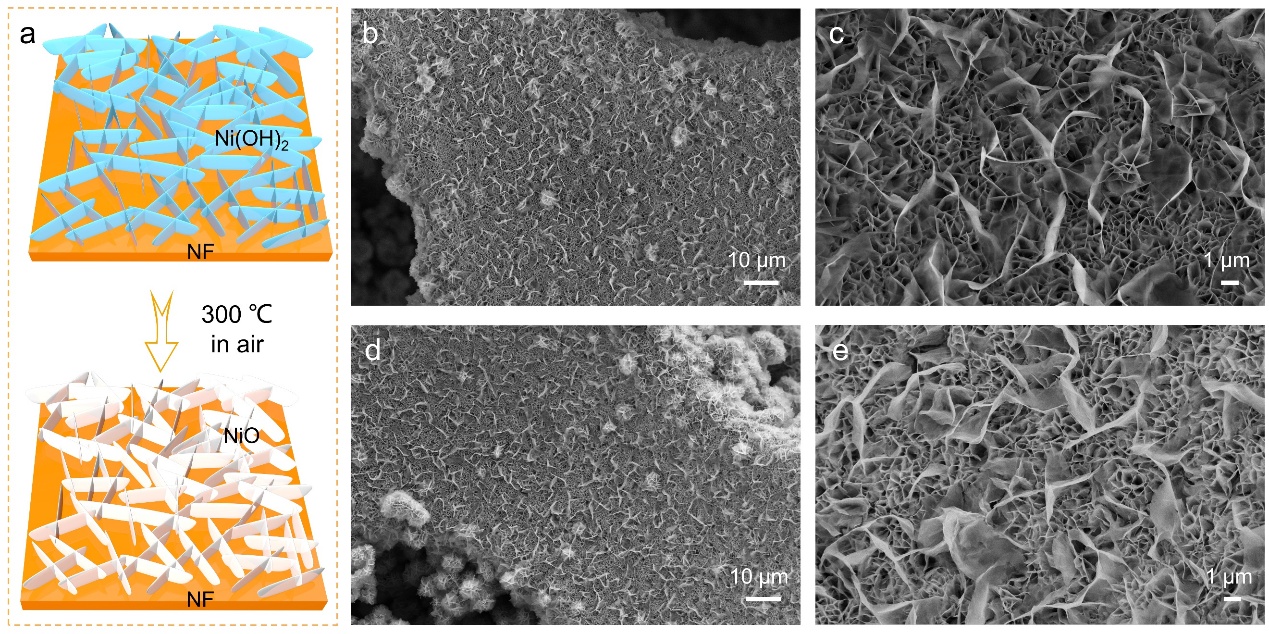


**Fig. S1** (a) Schematic diagram of the synthesis of NiO. (b-c) SEM of Ni(OH)_2_ on nickel foam (NF). (d-e) SEM of NiO on NF.

**Fig. S2** BET surface area of the Ni(OH)_2_ and NiO catalysts.


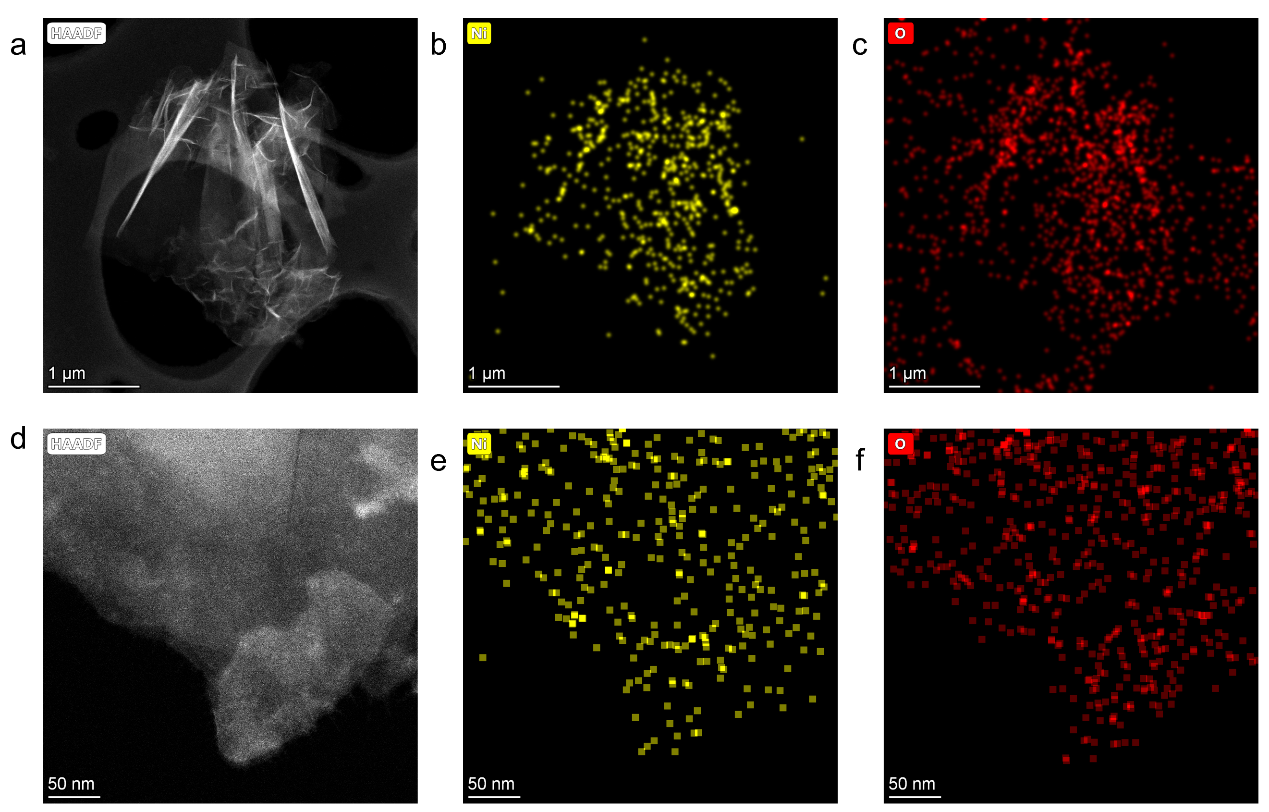


**Fig. S3** HADDF-STEM images of Ni(OH)_2_.


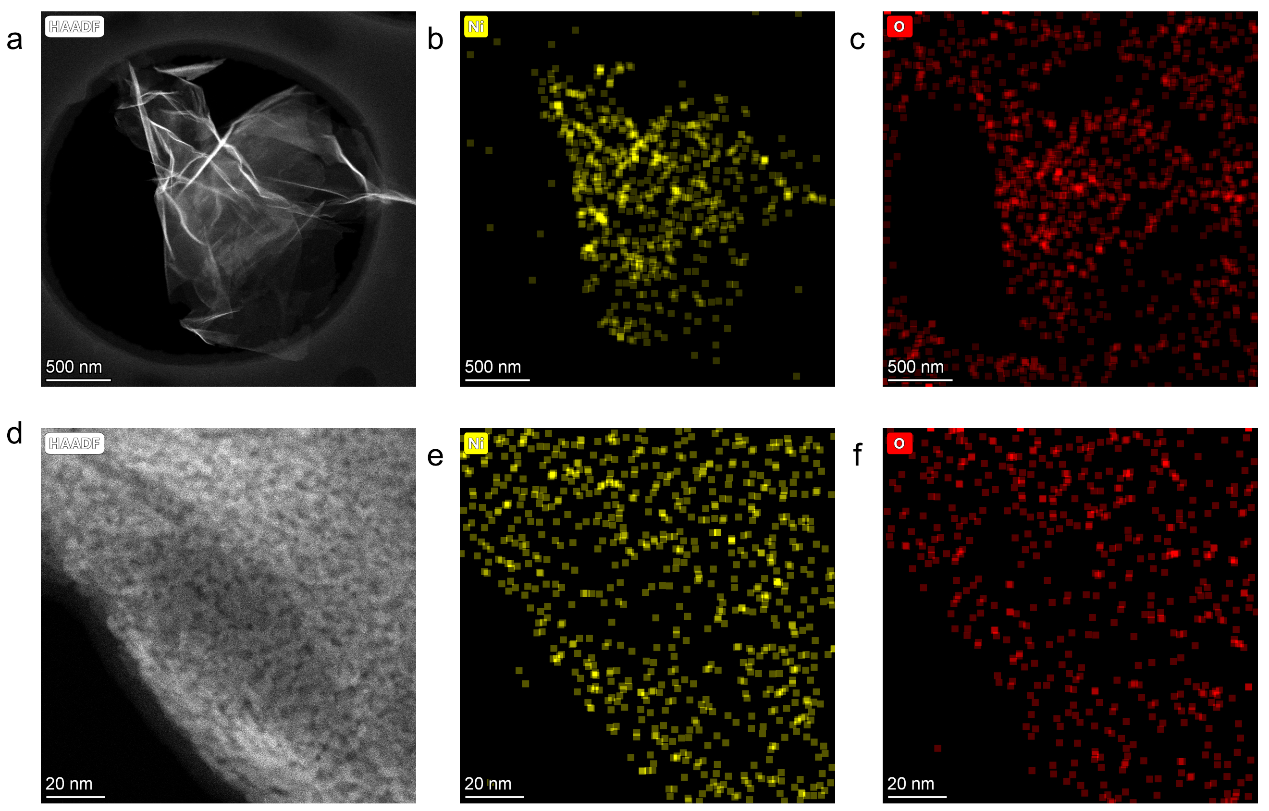


**Fig. S4** HADDF-STEM images of NiO.

**Fig. S5** Linear fitting of the anodic and cathodic peak current densities to the square roots of the scan rates for NiO (a) and Ni(OH)_2_ (b).

**Fig. S6** ECSA data for NiO and Ni(OH)_2_ in 1M KOH with 0.1 M glycerol. Cyclic voltammogram curves of (a) NiO and (b) Ni(OH)_2_. (c) C_dl_ of corresponding electrocatalysts.

**Fig. S7** The electrooxidation performance of other alcohols. LSV cures for ethylene glycol oxidation reaction (a), ethanol oxidation reaction (b), glycerol oxidation reaction(d), methanol oxidation reaction (e). LSV cures of alcohols oxidation reaction for NiO (c) and Ni(OH)_2_ (d).

**Fig. S8** In-situ EIS plots (Nyquist) of NiO and Ni(OH)_2_ at different reaction conditions. (a) EIS plots of NiO under GOR. (b) EIS plots of Ni(OH)_2_ under GOR. (c) EIS plots of NiO under OER. (d) EIS plots of Ni(OH)_2_ under OER.

**Fig. S9** Chronopotentiometric measurement of NiO in 1M KOH with 0.1 M glycerol.

**Fig. S10** High-resolution spectrum of Ni 2p (a), O 1s (b) and C 1s (c) of NiO before and after GOR, respectively.

**Fig. S11** High-resolution spectrum of Ni 2p (a), O 1s (b) and C 1s (c) of Ni(OH)_2_ before and after GOR, respectively.


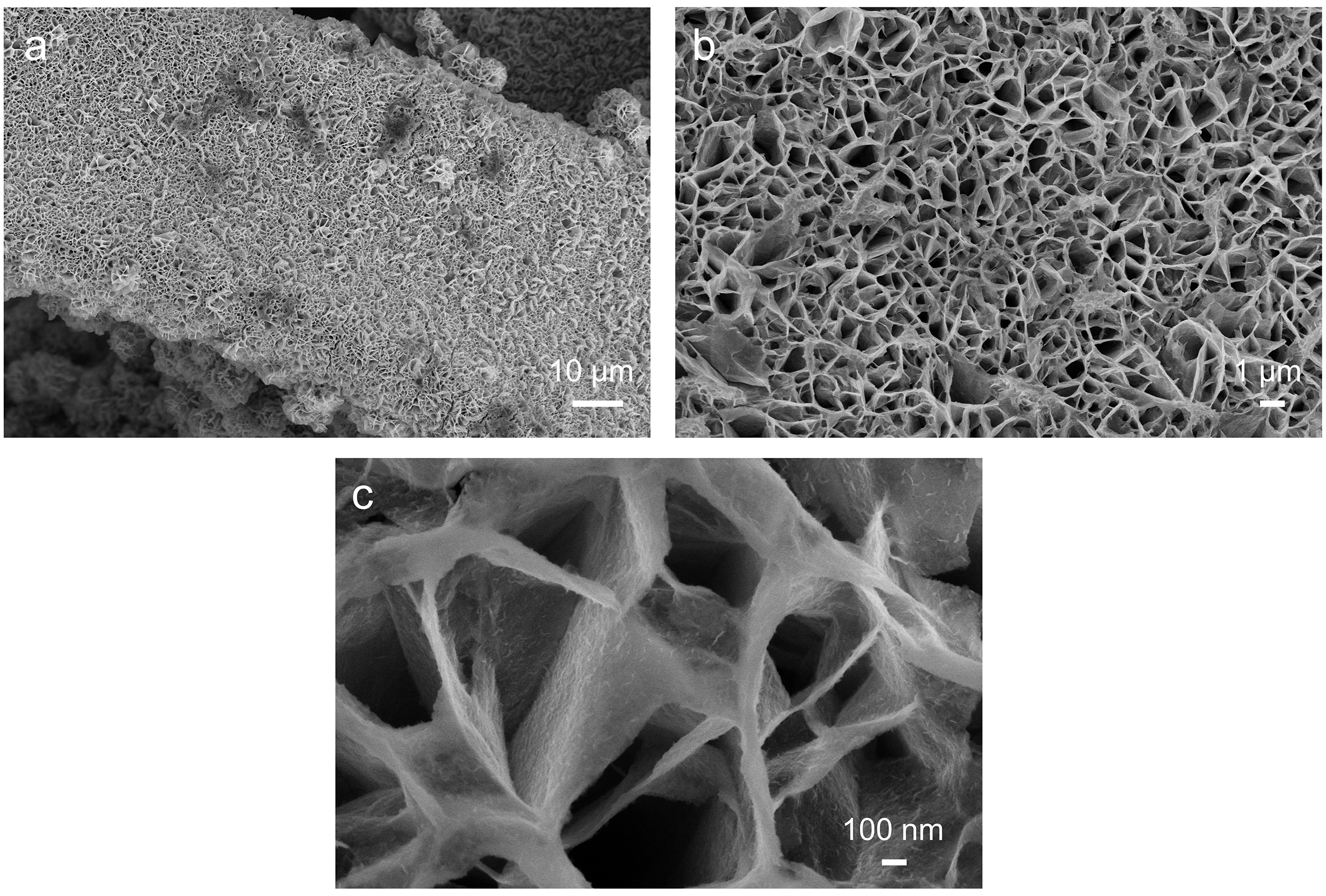


**Fig. S12** SEM images of NiO after GOR.

**Fig. S13** XRD pattern of NiO after GOR.


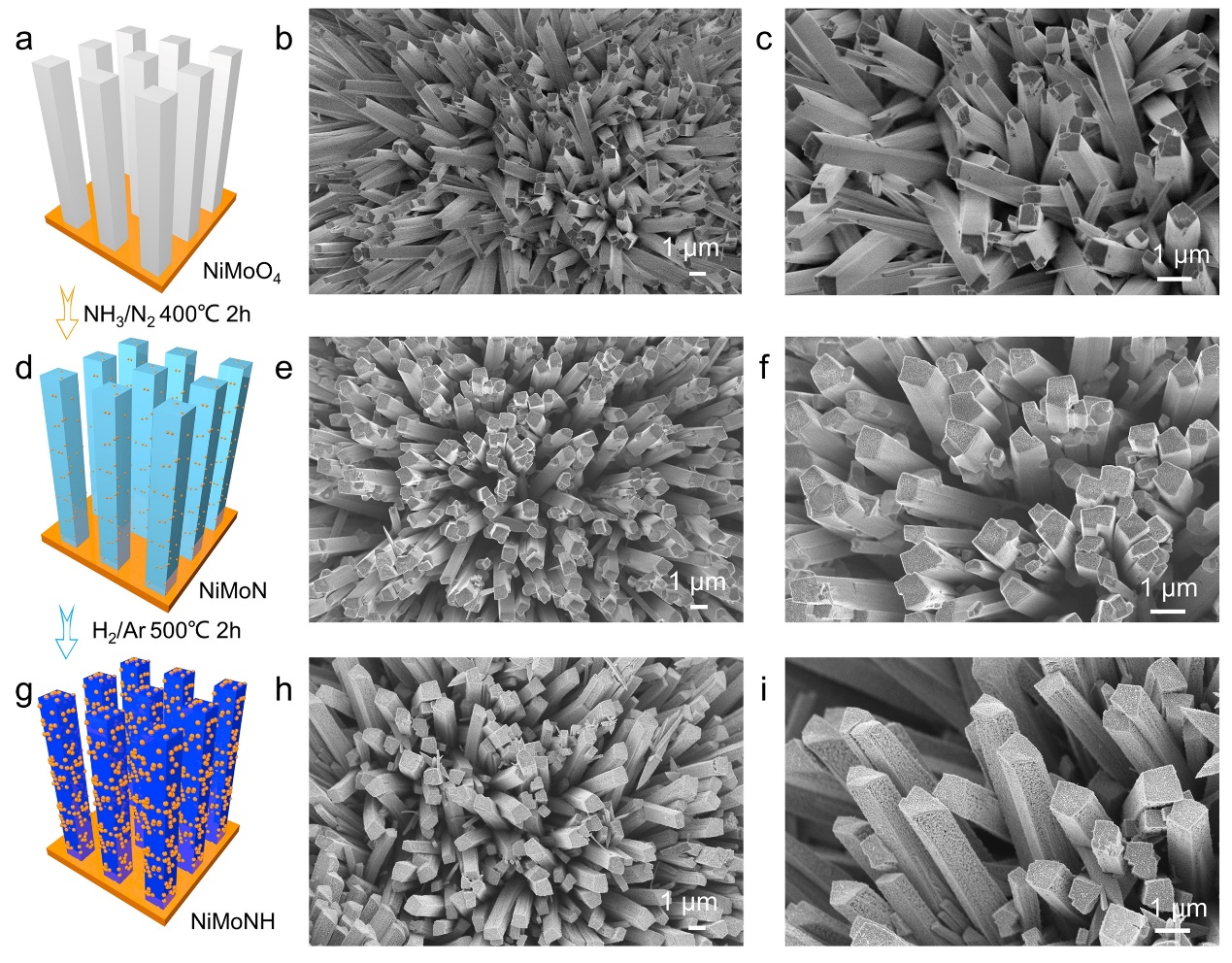


**Fig. S14** Schematic illustration and SEM images of NiMoO (a-c), NiMoN (d-f) and NiMoNH (g-i).


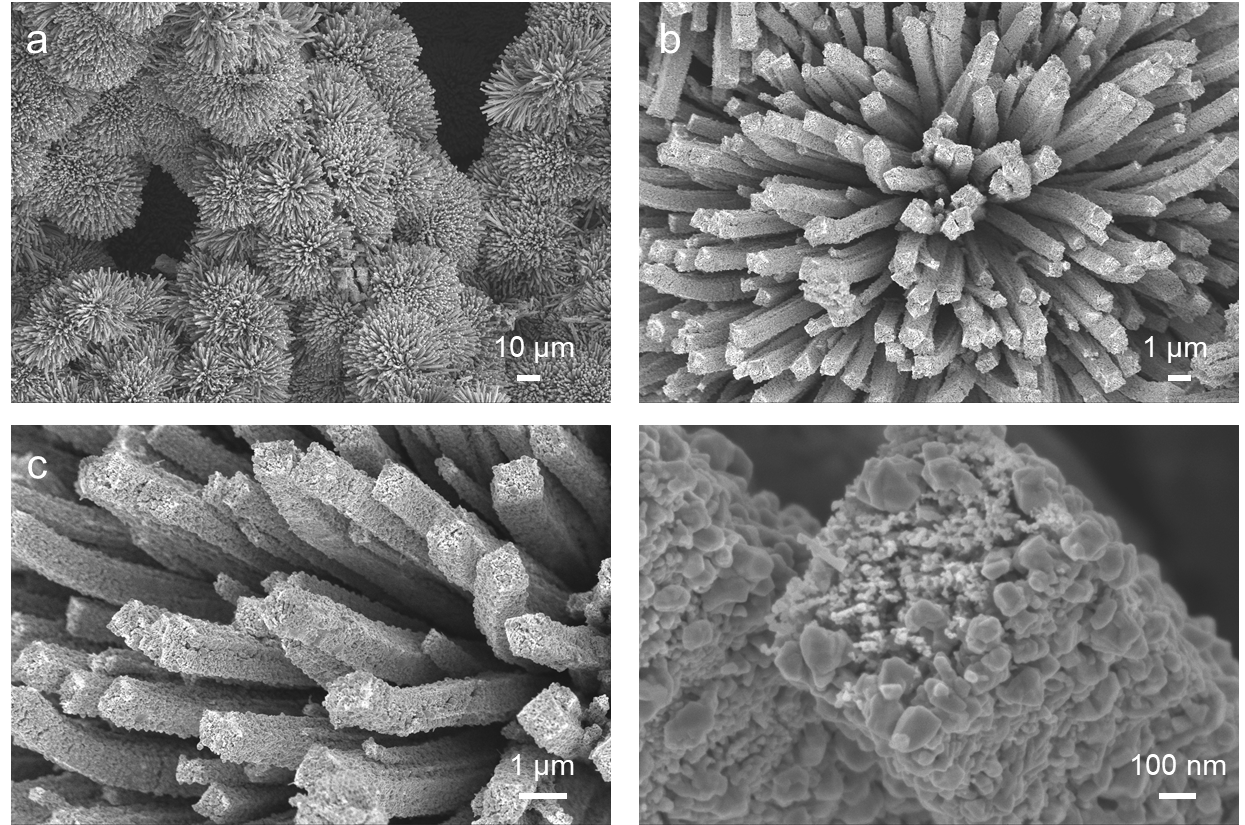


**Fig. S15** SEM images of NiMoH (a-d).


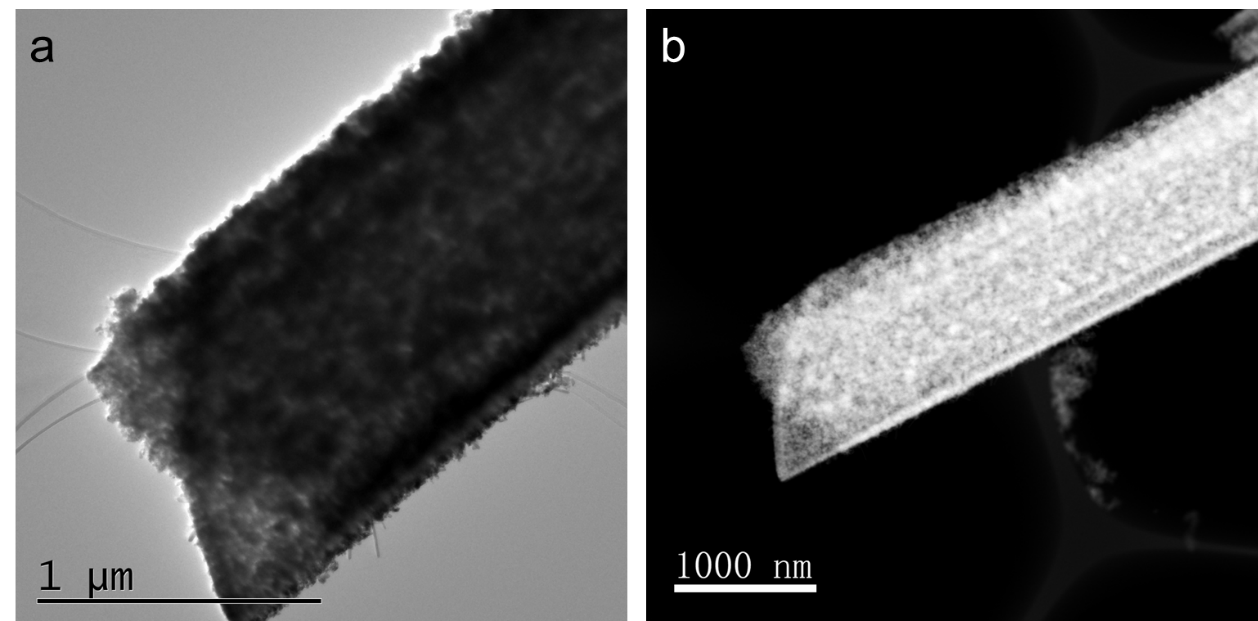


**Fig. S16** TEM (a) and HADDF-STEM (b) image of NiMoNH.

**Fig. S17** High-resolution N1s and Mo 3p XPS spectra of NiMoNH.


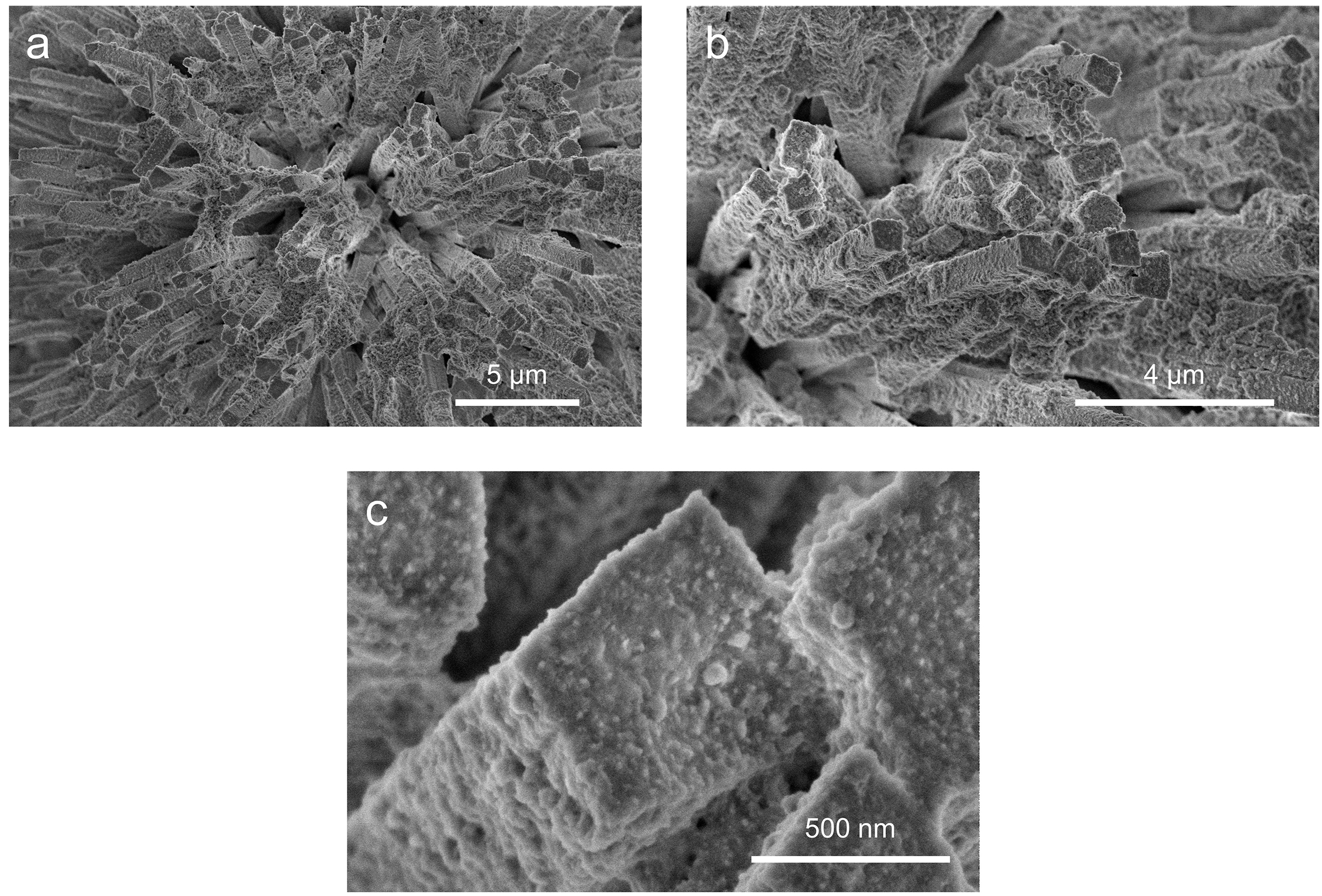


**Fig. S18** SEM images of NiMoNH after HER.

**Fig. S19** XRD pattern of NiMoNH after HER.

**Table S1**. The GOR activity of transition metal-based electrocatalysts.

| Electrocatalyst | Electrolyte | E_GOR_ ^a^ | E_OER_ ^b^ | ΔE ^c^ | Reference |
| --- | --- | --- | --- | --- | --- |
| NiO | 1.0 M KOH with and without 0.1 M glycerol | 1.3 | 1.59 | 293 | This work |
| R-NiCuO | 1.0 M KOH with and without 0.1 M glycerol | 1.2 | 1.53 | 330 | Energy Environ. Sci. 2022, 15, 3004. |
| CuCo-oxide | 0.1 M glycerol  in 0.1 M KOH | 1.25 | - | - | Adv. Mater. 2023, 35, e2203285. |
| CoNiCuMnMo-NPs/CC | 1.0 M KOH with and without 0.1 M glycerol | 1.25 | 1.55 | 300 | J. Am. Chem. Soc. 2022, 144, 7224. |
| NiO_x_/MWCNTs-Ox | 1.0 M KOH with and without 1 M glycerol | 1.31 | 1.59 | 280 | ACS Catal., 2022, 12, 982-992. |
| Ni–Mo–N/CFC | 1.0 M KOH with and without 0.1M glycerol | 1.39 | 1.67 | 280 | *Nat. Commun.*, 2019, 10, 5335. |
| CoNi film | 1.0 M KOH with and without 0.33 M glycerol | 1.3 | 1.6 | 300 | *J. Hydrogen Energy 2022, 47, 32145.* |

^a^ E_GOR_ vs RHE at 10 mA cm^-2^ during the GOR; ^b^ E_OER_ vs RHE at 10 mA cm^-2^ during the OER; ^c^ E_OER_-E_GOR_.

**Table S2.** The HER activity of NiMo-based Electrocatalysts.

| Electrocatalysts | Substrate | Electrolyte | Overpotential (mV) at 10 mA cm^-2^ | Tafel slope (mV dec^-1^) | Reference |
| --- | --- | --- | --- | --- | --- |
| MoO_2_-Ni | NF | 1 M KOH | 47 | 36.6 | ACS Catal. 2019, 9, 2275. |
| MoNi_4_/MoO_3−x_ | NF | 1 M KOH | 17 | 36 | Adv. Mater. 2017, 29, 1703311. |
| PS-MoNi@NF | NF | 1 M KOH | 30 | 37 | Adv. Energy Mater. 2021, 11, 2003511. |
| N-NiMoO_4_/NiS_2_ | carbon fiber cloth | 1 M KOH | 99 | 74.2 | Adv. Funct. Mater. 2019, 29, 1805298. |
| Ni_2_P/ MoO_2_/NF | NF | 1 M KOH | 34 | 45.8 | Appl. Catal., B 2020, 269, 118803. |
| N-NiMoS | NF | 1 M KOH | 68 | 86 | Appl. Catal., B 2020, 276, 119137. |
| Ni_0.2_Mo_0.8_N/Ni | NF | 1 M KOH | 14 | 33 | Energy Environ. Sci. 2020, 13, 3007. |
| MoNi_4_ | NF | 1 M KOH | 15 | 30 | Nat. Commun. 2017, 8, 15437. |
| NiMoNH | NF | 1 M KOH+0.1 M glycerol | 183 (100 mA cm^-2^) | 146 | This work. |

**Table S3.** Comparison of performance of small molecule oxidation-assisted hydrogen production.

| Catalyst | Electrolyte | Value-added  product | potential at 100 mA cm^-2^ | Reference |
| --- | --- | --- | --- | --- |
| hp-Ni\|\|hp-Ni | 10 mM Benzyl alcohol + 1M KOH | benzoic acid | 1.66 | ACS Catal. 2017, 7, 4564. |
| NiVRu-LDHs NAs/NF \|\|NiVRu-LDHs NAs/NF | 1 M KOH + 0.1 M glycerol | Formate | 1.62 | Adv. Mater. 2023, e2300935. |
| Mo-Ni\|\|Mo-Ni | 10 mM Benzyl alcohol + 1M KOH | benzoic acid | 1.53 | J. Mater. Chem. A 2019, 7, 16501. |
| Co_3_FeP_x_\|\|Co_3_FeP_x_ | 1 M KOH + 0.1 M  glucose | - | 1.59 | Appl. Catal., B 2020, 263, 118109. |
| MoO_2_-FeP@C\|\|MoO_2_-FeP@C | 10 mM 5-hydroxymethylfurfural + 1M KOH | 2,5-furandicarboxylic acid | 1.69 | Adv. Mater. 2020, 32, e2000455. |
| NiCo hydroxide \|\| NiCo hydroxide | 1 M KOH + 0.1 M glycerol | formate | 1.58 | Nat Commun 2022, 13, 3777. |
| NiMoNH\|\|NiO | 1 M KOH + 0.1 M glycerol | Formate | 1.54 | This work. |
